# Two rodent suborders have evolved missing amino acids in the lipid‐binding region of apolipoprotein E

**DOI:** 10.1002/lipd.12426

**Published:** 2025-01-13

**Authors:** Don L. Puppione

**Affiliations:** ^1^ Molecular Biology Institute, University of California Los Angeles California USA

**Keywords:** apolipoprotein E, *Castorimorpha*, *Hystricomorpha*, lipid‐binding region, *Rodentia*

## Abstract

The order Rodentia comprises nearly 45% of all extant taxa, currently organized into 31 living families, some 450 genera, and roughly 2010 species (Kelt & Patton, 2020). Considering that rodents began evolving at least 66 million years ago, it is not surprising that they have diversified into five distinct suborders. With the advent of molecular biology, this difference can often be seen at the molecular level as well. Previous studies have indicated that the apolipoprotein E (APOE) of guinea pigs, belonging to the suborder *Hystricomorpha*, have fewer amino acids than have been reported for other suborders of *Rodentia*. Searching the genomic database for hystricomorph *APOE* genes, it was found that hystricomorphs were missing residues both in the vicinity of the hinge region and in the lipid‐binding region of the apolipoprotein. In the hinge region, missing residues varied between 5 and 3, and in the latter region, seven residues were missing. The search also revealed that castorimorphs, although lacking the smaller of the two deletions, were also missing the same seven residue deletion as found in APOE of the hystricomorphs.

## INTRODUCTION

Apolipoproteins have been shown to play multiple roles in lipid and lipoprotein metabolism in primates, rodents, and other mammals. Many of these studies focused on the inability of mammals to tolerate diets high in triglycerides and cholesterol. Among rodents, dietary cholesterol was found to be extremely lethal to the guinea pig (*Cavia porcellus*). The seminal studies on the University of California campus in Berkeley were carried out by Ruth Okey and colleagues in the 1930s and 1940s, and then continued later by her colleague Rosemarie Ostwald (Okey, 1942; Okey, 1944; Okey & Greaves, 1939; Ostwald et al., 1966). Initially, Okey and Greaves (1939) found that feeding of cholesterol resulted in the animals developing enlarged liver and spleen, forming gallstones and becoming anemic. In addition, the level of unesterified cholesterol increased in the plasma. This latter observation was later shown to have a marked effect on the lipoproteins in the HDL fraction (Puppione et al., 1971; Sardet et al., 1972). Guinea pigs on a control diet had no detectable HDL based on measurements taken with the analytical ultracentrifuge; however, they were detected in the cholesterol‐fed animals (Puppione et al., 1971). Compositional studies showed that the increase in concentration was associated with HDL becoming enriched in unesterified cholesterol, not cholesteryl esters, as had been shown to be the case in other mammals (Puppione et al., 1971; Sardet et al., 1972). Subsequent electron microscope studies showed that HDL of cholesterol‐fed guinea pigs were discoidal in shape, whereas typical HDL are spheroidal (Guo, Hamilton, Kane, et al., 1982; Guo, Hamilton, Ostwald, & Havel, 1982; Sardet et al., 1972). When the plasma apolipoproteins were measured, it was found that APOE had increased 10‐fold when on the diet for 1 week and 22‐fold after 18–22 weeks on the diet (Guo, Hamilton, Kane, et al., 1982; Guo, Hamilton, Ostwald, & Havel, 1982).

Later, when the apolipoprotein E gene of the guinea pig was sequenced, the encoded protein was found to be short, having 280 amino acids as compared with 299 in the human ortholog (Matsushima et al., 1990). Alignment showed that 5 and 7 amino acids were missing in two different locations in the C‐terminal region of apoE. Another report, looking at the evolution of mammalian apoE, noted that two other rodents, house mouse (*Mus musculus*) and brown rat (*Rattus norvegicus*), were not missing amino acids in these two regions (Yang et al., 1991). More recently, the naked mole‐rat (*Heterocephalus glaber*) and the degu (*Octodon degus*), like the guinea pig, were also reported to have smaller APOE (Hurley et al., 2022; Puppione et al., 2021).

The guinea pig, the naked mole‐rat, and the degu are different from the mouse and the rat due to variation in the mastication apparatus and the structure of the mandible (Brandt, 1855). In the past, these characteristics were the basis for separating rodents into different suborders that evolved separately. The mammalian order *Rodentia* currently has been divided into five suborders: *Hystricomorpha*, *Myomorpha*, *Castorimorpha*, *Sciuromorpha*, and *Anomaluromorpha* (Flynn et al., 2019; Kelt & Patton, 2020). Guinea pigs, naked mole‐rats, and degus are hystricomorphs, and mice and rats are myomorphs. Molecular evidence now has been accumulated from numerous studies (Adkins et al., 2001; Bininda‐Emonds et al., 2007; Blanga‐Kanfi et al., 2009; D'Elía et al., 2019; Huchon et al., 2000, 2007; Montgelard et al., 2008) that better distinguish rodent division.

Based on the above mentioned reports on the differences in rodent APOE, a search of the National Center for Biotechnology Information (NCBI) genomic database was carried out for different rodent apolipoprotein E gene to see whether other differences could be found. Consistent with what had been reported previously, all hystricomorphs, except for the gundi (*Ctenodactylus gundi*), were found to be similar to the guinea pig. Upon aligning with the human sequence, those belonging to the infraorder *Hystricognathi* had two deletions in the C‐terminal region of APOE, with one varying between 5 and 3 missing residues and the other consisting of seven missing residues. But the gundi, belonging to the suborder *Ctenodactylomorphi*, was missing 11 residues, located between the two gaps seen among the hystricognathus rodents. Interestingly, the castorimorphs were also found to have a deletion of seven residues and in the same location as the hystricognathous rodents when alignment was done with the human ortholog. These deletions were not found in other rodents. We report here on the location of these deletions in 21 hystricomorphs and 7 castorimorphs. Based on previous studies in other mammals, a lipid‐binding region has been identified in the C‐terminal region of APOE (Chen et al., 2011; Marais, 2019). When aligned with human APOE4, the seven residue deletions of both castorimorphs and hystricomorphs were found to be located within a 32 amino acid sequence, considered to be the lipid‐binding region of human APOE, residues 241–272 (de Lima Pizzico et al., 2024).

## METHODS

### Locating rodent apoE genes in the NCBI genomic database

Using the DNA sequence for the *APOE* gene of the house mouse (*M. musculus*; ENSMUST00000174064.80), obtained from the University of California at Santa Cruz genome browser (Raney et al., 2024), it was possible to find the various rodent genes, using a combination of NCBI Basic Local Alignment Search tool and the DNA to protein translator of the University of the Basque Country (www.insilico.ehu.es).

### Alignment of the encoded APOE sequences

**FIGURE 1:**
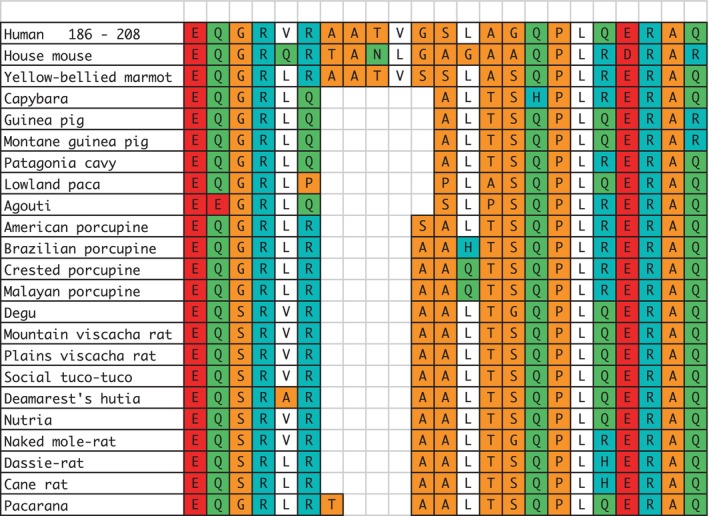
Rodent APOE sequences aligned with a segment of the C‐terminal region of human APOE4 between residues 186 and 203. Background colors indicate the relative polarity of the amino acids: Negative: D, E (red); positive: R, K, H (blue); polar: N, Q (green); neutral: A, G, S, P, T (tan); hydrophobic: F, I, L, M, V (white).

**FIGURE 2:**
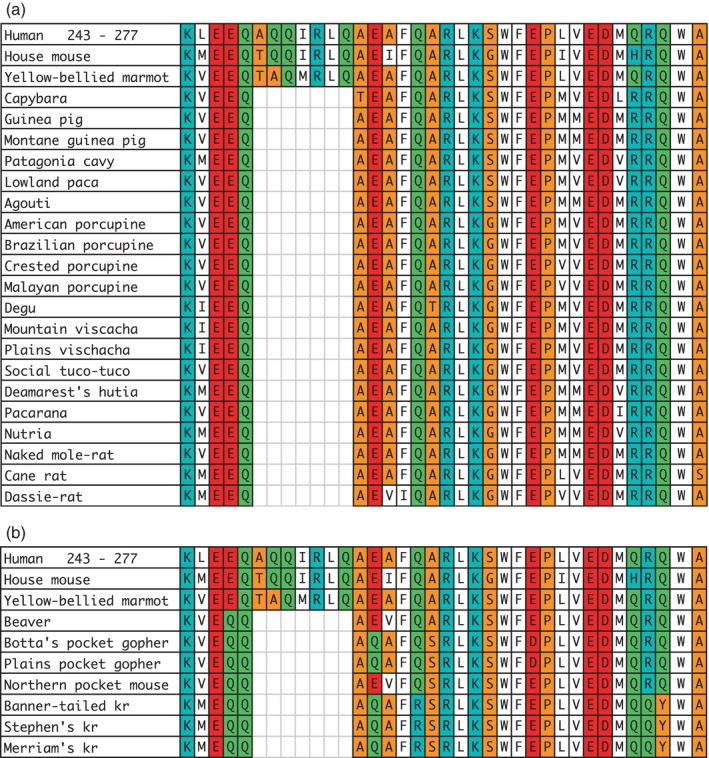
Rodent APOE sequences aligned with a segment of the C‐terminal region of human APOE4 between residues 243 and 277. Background colors indicate the relative polarity of the amino acids, as shown in Figure 1. (a) Hystricomorphs sequences are shown. (b) Castorimorph sequences are shown. kr, kangaroo rat.

The APOE amino acid sequences were obtained after exons 2, 3, and 4 were located. Sequences obtained for the castorimorphs and hystricomorphs were aligned with human APOE4. Rodent APOE, like human APOE4, does not contain any cysteines in the mature apolipoprotein. The complete human sequence of APOE4 is shown in Table 1. The alignments where gaps were observed are shown in Figures 1 and 2. Included in these figures are the corresponding segments obtained from the APOE sequences of the house mouse (P08226) and the yellow‐bellied marmot (*Marmota flaviventris*) (see Table 1). Alignments of the sequences were obtained using Clustal Ω available on the webpage of Kyoto University Bioinformatics Center, Kyoto, Japan (bic.kyoto-u.ac.jp). The following information for each rodent listed in both figures can be seen in Table 1: the common name, the taxonomic name, and the accession number of the genomic listing and the calculated molecular weight.

**TABLE 1:** Encoded rodent apoE sequences.

	Hystricomorpha
Ctenodactylomorphi	
Ctenodactylidae	**Gundi** (*Ctenodactylus gundi*; PVKB01000401)
	32074 Da
	MKILWAVLTV TFLAG CQA
	EVEPEVEPEV RDPAGWQAGQ PWELALGRFW DYLRWVLTLS DQVQEELLSS QVTQELT
	ALMEETMQEL KAYRSELEQQ LGPMAEETRA RLTKELQAAQ ARLGADMEDA RGRLAQYRGE
	VQAMLGQSAE EMRARLASHL RKLRKRLLRD ADDLHARLAV YRAGAREGVE RGVGAMRERL
	GPLLEQGRLR AVSVGARSAP PLQERAQAWG ERLR‐ ‐ ‐ ‐ ‐ ‐ ‐ ‐ ‐ ‐ ‐EAGAR AREQADAMRA
	AMEEQAEQVR LQAEAFQARL RSWFEPVVED MQHKWAEFVE KVQVAVGAGT TAAPQEAP
Hystricognathi	
Hystricidae	**Crested porcupine** (*Hystrix cristata*; PVJO010000840)
	32937 Da
	MKVLWAVLVV TLLAG CQA
	DVEPALEVGE PAPEVREPAM WQSGQPWELA LGRFWDYLRW VQTLSDQVQE ELLSSQVTQE LT
	VLMEDTMKEV KAYKSELEQE LGPMAEDTKA RLSKELQAAQ ARLGADMEEV RNRLTQYRSE
	VQTMLGQSAE ELRARLASHL RKLRKRLLRD AEDLQKRLAV YKAGAQEGAE RGVSAIRERL
	GSLVEQGRLR ‐ ‐ ‐ ‐AAQTSQ PLRERAQAWG ERLRGRLEEV GGQARDRLDV VREQMEEVRA
	KVEEQ‐ ‐ ‐ ‐ ‐ ‐ ‐AEAFQARL KGWFEPVVED MRRQWAELIE KVQVAVGAST PAPSEKH
	**Malayan porcupine** (*Hystrix brachyura*; QZML01001391)
	32937 Da
	MKVLWAVLVV TLLAG CQA
	DVEPALEVGE PAPEVREPAM WQSGQPWELA LGRFWDYLRW VQTLSDQVQE ELLSSQVTQE LT
	VLMEDTMKEV KAYKSELEQE LGPMAEDTKA RLSKELQAAQ ARLGADMEEV RNRLTQYRSE
	VQTMLGQSAE ELRARLASHL RKLRKRLLRD AEDLQKRLAV YKAGAQEGAE RGVSAIRERL
	GSLVEQGRLR ‐ ‐ ‐ ‐ AAQTSQ PLRERAQAWG ERLRGRLEEV GGQARDRLDV VREQMEEVRA
	KVEEQ‐ ‐ ‐ ‐ ‐ ‐ ‐AEAFQARL KGWFEPVVED MRRQWAELIE KVQVAVGAST PAPSEKH
Hydrochaeridae	**Capybara** (*Hydrochoerus hydrochaeris*; PVLA01002003)
	32330 Da
	MKILWAALVL TLLAG CRA
	DVEPEVEVRE TAVWQSGQPW ELALSRFWDY LRWVQTLSDQ VQEELLSSQV TQELT
	LLMEDTMKEL KAYKSELEKE VGPMAEDTKA RLSKELQGAQ ARLAGDMEEV RNRLSQYRSE
	VQAMLGQSSE ELRARLASHL RKLRKRLQRD AEELQKRLAV YKAGAQEGAE RGVSAIRERL
	GSLMEQGRLQ ‐ ‐ ‐ ‐ ‐ALTSH PLRERAQAWG EQVRGRLEKV GSQARDRLEE VREQMEEVRV
	KVEEQ‐ ‐ ‐ ‐ ‐ ‐ ‐TEAFQARL KSWFEPMVED LRRQWAELIE KVQVAVGAST SPPSQKS
Caviidae	**Guinea pig** (*Cavia porcellus*; AAKN02048232)
	32404 Da
	MKVLWAALVV TLLAG CRA
	DVEPEVEVRE PAVWQSGQPW ELALSRFWDY LRWVQTLSDQ VQEELLSNQV TQELT
	LLIEDTMKEV KDYKAELEKE LGPVAEDTKA RLAKELQAAQ ARLGADMEEV RNRLSQYRSE
	VQAMLGQSSE ELRARLTSHL RKMRKRLQRD IDELQKRMAV YKAGAQEGAE RGVSAIRERL
	GSLIEQGRLQ ‐ ‐ ‐ ‐ ‐ALTSQ PLQERAQAWG EQMRGRLEKV GSQARDRLEE VREQMEEVRV
	KVEEQ‐ ‐ ‐ ‐ ‐ ‐ ‐AEAFQARL KSWFEPMMED MRRQWAELIQ KVQVAVGAST SAPSQEP
	**Montane guinea pig** (*Cavia tschudii*; PVKK010002674)
	32404 Da
	MKVLWAALVV TLLAG CRA
	DVEPEVEVRE PAVWQSGQPW ELALSRFWDY LRWVQTLSDQ VQEELLSNQV TQELT
	LLIEDTMKEV KDYKAELEKE LGPVAEDTKA RLAKELQAAQ ARLGADMEEV RNRLSQYRSE
	VQAMLGQSSE ELRARLTSHL RKMRKRLQRD IDELQKRMAV YKAGAQEGAE RGVSAIRERL
	GSLIEQGRLQ ‐ ‐ ‐ ‐ ‐ALTSQ PLQERAQAWG EQMRGRLEKV GSQARDRLEE VREQMEEVRV
	KVEEQ‐ ‐ ‐ ‐ ‐ ‐ ‐AEAFQARL KSWFEPMMED MRRQWAELIQ KVQVAVGAST SAPSQEP
	**Patagonian cavy** (*Dolichotis patagonum*; PVJX010013916)
	32317 Da
	MKILWAALVV TLLAG CQA
	DVEPEVEVRE PAVWQSGQPW ELALGRLWDY LRWVQTLSDQ VQEELLSSKV TQELT
	LLMEDTMKEV TAYKSELEKE LGPMAEDTKA RLSKELQGAQ ARLGADMEEV RNRLLQYRSE
	VQAMLGQSSE ELRARLASHL RKLRKRLQRD ADDVQKRLAV YRAGAQEGAE RSVSAIRERL
	GSLMEQGRLQ ‐ ‐ ‐ ‐ ‐ALTSQ PLRERAQAWG EQMRGRLEKV GSQARDRLDE VREQMEEVRV
	KMEEQ‐ ‐ ‐ ‐ ‐ ‐ ‐AEAFQARL KSWFEPMVED VRRQWAELMQ KVQVAMGAST PAPSQKP
Dasyproctidae	**Agouti** (*Dasyprocta punctata*; RJWM01082531)
	32474 Da
	MKVLWAALVV TLLAG CQA
	DVEPELEVQE PAVWQSGQPW ELALGRFWDY LRWVQTLSEQ VQEELLSSHV TQELT
	LLMEDTMKEV KAYKSELEQE LAPMAEDTKA RLSKELQAAQ SRLRADMEEV LNRLSQYRGE
	VQTMMGQSGE ELRARLAAHL RKLRKRLLRD VEEVQKRMDV YRAGAQEGAE RSVSAIRERM
	GSLVEEGRLQ ‐ ‐ ‐ ‐ ‐SLPSQ PLRERAQAWG EQMRGRLEKV GSQARDRLEE VREQMEEVRG
	KVEEQ‐ ‐ ‐ ‐ ‐ ‐ ‐AEAFQARF KSWFEPMMED MRRQWADLIE KVQVAVGAST AAPSQKS
Cuniculidae	**Lowland paca** (*Cuniculus paca* paca; RJWT010052400)
	31746 Da
	MKLLWAALVV TLLAG CRA
	DVEPELEMQE PAVWQSGQPW ELALGRFWDY LRWVQTLSDQ VQEELLSSQV TQELT
	LLMEDTMKEV KVYKSKLEEE LAPMAEETKA RLSKELQAAQ ARLGADMEEV RNRLSQYRGE
	VQAVLGQSAD ELRARLASHL RKLRKRLLRD AEDLQKRLAV YKAGAQEGAE RGVSAIRERL
	ESLAEQGRLP ‐ ‐ ‐ ‐PLASQ PLQERAQAWG EQVRGRLERV GSQARDRLEE VREQMEEVRV
	KVEEQ‐ ‐ ‐ ‐ ‐ ‐ ‐AEAFQARL KSWFEPMVED VRRQWADLIE KVQQAVGTSQ KA
Erethizontidae	**American porcupine** (*Erethizon dorsatum*; SWEC01025652)
	32153 Da
	MKVLWAVLVV TLLAG CRA
	DAEPELEAQE PAVWQSGQPW ELALGRLWDY LRWVQTLSDQ VQEELLSSQV TQELT
	LLMEDTMKEV KAYKAELEQE LAPMAEDTRA RLSKELQAAQ ARLGADMEEV RNRLAQYRGE
	VQAMLGQSAE ELRARLASHL RKMRKRLLRD AEDLQKRLAV YKAGAREGAE RGVSAIRERL
	ASLVEQGRLR ‐ ‐ ‐ ‐SALTSQ PLRERAQAWG ERLRGRLEEV GGQARDRLDV VREQMEEVRA
	KVEEQ‐ ‐ ‐ ‐ ‐ ‐ ‐AEAFQAR LKGWFEPMVE DMRRQWADLI EKVQVAVGAS TPAPSQKP
	**Brazilian porcupine** (*Coendou prehensilis*; JAPYYN010000031)
	32147 Da
	MKVLWAVLVV TLLAG CRA
	DAEPELEAQE PAVWQSGQPW ELALGRLWDY LRWVQTLSDQ VQEELLSSQV TQELT
	LLMEDTMKEV KAYKAELEQE LAPMAEDTRA RLSKELQAAQ ARLGADMEEV RNRLAQYRGE
	VQAMLGQSAE ELRARLASHL RKMRKRLLRD AEDLQKRLAV YKAGAREGAE RGVSAIRERL
	GSLVEQGRLR ‐ ‐ ‐ ‐AAHTSQ PLRERAQAWG ERLRGRLEEV GGQARDRLDV VREQMEEVRA
	KVEEQ‐ ‐ ‐ ‐ ‐ ‐ ‐AEAFQARL KGWFEPMVED MRRQWADLIE KVQVAVGAST PAPSQKP*
Octodontidae	**Degu** (*Octodon degus*; AJSA01200869)
	32277 Da
	MKVLCTVLVV TLLAG CRA
	DVEPEPEVLE PAVWQSGQPW ELALGRLWDY VRWVQTLSDQ VQEELLSSQV TQELT
	VLMEDTMKAV KAYKSELEQE LVPMAEDTKA RLSKELQAAQ ARLGADMEEV RNRLAQYRNE
	MQAMLGQSAD ELRARLASHL RKLRKRMLRD AEDLQKRLAV YKDGASEGAE RGVSAIRERL
	GSLVEQSRVR ‐ ‐ ‐ ‐AALTGQ PLQERAQAWG KQLRGRLEEV RGQAQDRLEE MREQMEEVRV
	KIEEQA‐ ‐ ‐ ‐ ‐ ‐AEAFQTRL KGWFEPMVED MRRQWADLIE KVQAAVGAST PVPSQKP
	**Mountain viscacha rat** (*Octomys mimax*; NDGM011023505^a^)
	32216 Da
	CQA
	DVEPEPEVLE PAVWQSGQPW ELALGRLWDY VRWVQTLSDQ VQEELLSSQV TQELT
	VLMEDTMKAV KAYKSELEQE LVPMAEDTKA RLSKELQAAQ ARLGADMEEV RNRLALYRNE
	MQAMLGQSAE ELRARLASHL RKLRKRMLRD AEDLQKRLAV YKDGASEGAE RGVSAIRERL
	GSLVEQSRVR ‐ ‐ ‐ ‐AALTSQ PLQERAQAWG KQLRGRLEEV RGQAQDRLEE VREQMEEVRV
	KIEEQ‐ ‐ ‐ ‐ ‐ ‐ ‐AEAFQARL KGWFEPMV EDMRRQWADL IEKVQAAVGA STPAPTQNP
	**Plains vischaca rat** (*Tympanoctomys barrerae*; NDGN011413885)
	32250 Da
	MKVLCTVLVV TLLAG CRA
	DVEPEPEVLE PAVWKSGQPW ELALGRFWDY VRWVQTLSDQ VQEELLSSQV TQELT
	VLMEDTMKAV KAYKSELEQE LVPMAEDTKA RLSKELQAAQ ARLGADMEEV RNRLALYRNE
	MQAMLGQSAE ELRARLASHL RKLRKRMLRD AEDLQKRLAV YKDGASEGAE RGVSAIRERL
	GSLVEQSRVR ‐ ‐ ‐ ‐AALTSQ PLQERAQAWG KQLRGRLEEV RGQAQDRLEE VREQMEEVRV
	KIEEQ‐ ‐ ‐ ‐ ‐ ‐ ‐AEAFQARL KGWFEPMVED MRRQWADLIE KVQAAVGAST PAPTQNP
Ctenomyidae	**Social tuco‐tuco** (*Ctenomys sociabilis*; PVKA01010304)
	32300 Da
	MKVLCTVLVV TLLAG CQA
	DVQPEPEALE PAVRKSDQPW ELALGRFWDY LRWVQTLSDQ VQEELLSSQV TQELT
	VLMEDTMKAV KAYKSELEQE LVPMAEDTKA RLSKELQAAQ ARLGADMEEV RNRLAQYRSE
	MQAMLGQSAE ELRARLASHL RKLRKKLLRD AEDLQKRLAV YKDGASEGAE RSVSAVRERL
	ESLVEQSRAR ‐ ‐ ‐ ‐AALTSQ PLQERAQAWG KRLRGRLEEV GSQARDRLEE VREQMEEVRV
	KMEEQ‐ ‐ ‐ ‐ ‐ ‐ ‐AEAFQARL KGWFEPMVED MRRQWADLIE KVQAAVGAST PTPSQKP
Capromyidae	**Desmarest's hutia** (*Capromys pilorides*; PVKN010242851^a^)
	32321 Da
	CRA
	DVQPESEALE QAVWKTGQPW ELALGRFWDY LRWVQTLSDQ VQEEILSSQV TQELT
	VLIEDTMKAV KAHKSELEQE LVPMAEDTKA RLSKELQAAQ ARLGADMEEV RNRLAQYRSE
	MQAMLGQSAE ELRTRLASHL RKLRKRLLRD AEDLQKRLEV YKNGASEGAE RGVSAIRERL
	GSLVEQSRVR ‐ ‐ ‐ ‐AALTSQ PLRERAQAWG ERLRGRLEEV GGQARDRLDE VREQMEEVRI
	KMEEQ‐ ‐ ‐ ‐ ‐ ‐ ‐AEAFQARL KGWFEPM MEDVRRQWADL IEKVQAAVG TSTTAPSQKP
Echimyidae	**Nutria** (*Myocastor coypus*; PVJA010084934)
	32786 Da
	MKVLCTVLVV TLLAG CRA
	DVEPEPEALE PAVWKTGQPW ELALGRFWDY LRWVQTLSDQ VQEELLSSQI TQELT
	VLMEDTMKAV KAYKSELEQE LVPMAEDTRA RLSKELQAAQ ARLGADMEEV RNRLAQYRSE
	VQAMLGQSTE ELRGRLSSHL RKLRKRLLRD AEDLQKRLAV YKDGASEGAE RGVSAIRERL
	ERLGSLVEQS RVR‐ ‐ ‐ ‐AAL TSQPLRERAQ AWSERLRGRL EEVGGQARDR LEEVREQMEE
	VRVKMEEQ‐ ‐ ‐ ‐ ‐ ‐ ‐AEAFQ ARLKGWFEPM MEDVRRQWAD LIEKVQAAMS TSTPAPSQKP
Dinomyidae	**Pacarana** (*Dinomys branickii*; PVLD010006399)
	32436 Da
	MKVLWAVLVV TLLAG CQA
	DVEPELEAQE PAVWQNGQPW ELALGRFWDY LRWVQTLSDQ VQEELLSSQV TQELT
	VLMEDTMKEV KAYKSELEQE LAPMAEETKA RLSKELKAAQ ARLGADMEEV RNRLSQYRGE
	VQSMLGHSAE ELRARLATHL RKLRKRLLRD AEDLQKRLAV YKAGASEGAE RSVSAIRERL
	GSLVEQGRLR T‐ ‐ ‐AALTSQ PLQERAQAWG ERLRGRLEEV GSKARDRLDE VREQMEEVRL
	KVEEQ‐ ‐ ‐ ‐ ‐ ‐ ‐AEAFQARL KGWFEPMMED IRRQWADLIE KMQAAVGTST PAPTQK
Heterocephalidae	**Naked mole‐rat** (*Heterocephalus glaber*; RPGA01000009)
	32525 Da
	MKALWAVLVV TLLAG CRA
	DVQPELEMQE PALWQSGQPW ELALGRFWDY LRWVQTLSDQ VQEELLNSQV TQELT
	VLMEDTMKEV KAYKNELEEE LGPVAEDTKA RLSKELQGAQ ARLRADMEEV RNRLAHYSEE
	MQVMLGQSPD ELRARLGSHL RKLRKRLLRD AEDLQKRLAV YKAGAREGAE RGVSAIRERL
	GSLVEQSRVR ‐ ‐ ‐ ‐AALTGQ PLRERAQAWG ERLRGRLEEV GGRARDRLDE VREQMEEVRA
	KVEEQ‐ ‐ ‐ ‐ ‐ ‐ ‐ AEAFQARL KGWFEPMMED MRRQWADLIE KVQLAVGAST PVPSEDH
Petromuridae	**Dassie‐rat (** *Petromus typicus*; PVIR01048187)
	32183 Da
	MKSLWAVLVI TLLAG CQA
	DVQPELDVQV PAGWQGEKPW ELALGRFWDY LRWVQTLSDQ VQEELVNSQV TQELT
	SLMEDTMKEV KAYKSRLEQE VGPVAEDTKA RLSKELQAAQ ARLGADMEEV RDRVAQYRSE
	IQAMLGQSTE ELRARLASHL RKLRKRLLRD AEDLQKRLAV YKAGAQEGAE RGVGAIRERL
	GSLVEQSRLR ‐ ‐ ‐ ‐AALTSQ PLHERAQAWG ERLRGRLEEV GGRARDRLDE VREQVEEVRA
	KMEEQ‐ ‐ ‐ ‐ ‐ ‐ ‐ AEVIQARL KGWFEPVVED MRRQWADFIE KVQAAVGAST TVPSESR
Thryonomyidae	**Greater cane rat** (*Thryonomys swinderianus*; PVIC010012398)
	32283 Da
	MKVLWALLVV TILAG CRA
	EVQPELEVQA PAGWQSGQPW ELALGRFWDY LRWVQTLSDQ VQEELLNSQV TQELT
	VLMEDTMKEV KAYKSELEQE LGPMAEDTKA RLSKELQAAQ ARLGADMEEV RNRLAQYRGE
	MQAMLGQSAE ELRARLASHL RKLRKRLLRD AEDLQKRLAV YRAGAQEGAE RGVSAIRERL
	GSLVEQSRLR ‐ ‐ ‐ ‐ AALTSQ PLHERAQAWG ERLRGRLEEV GGQARDRLDE VREQMQEVRA
	KMEEQ‐ ‐ ‐ ‐ ‐ ‐ ‐AEAFQARL KGWFEPLVED MRRQWSDLVE KVQVAVRAST AAPSENH
Castorimorpha	
Castoridae	**American beaver** (*Castor canadensis*; RPDE01002880)
	32701 Da
	MNALWTVLVV TLLAG CQA
	DVEPQLEPEV QEQAGWQTGQ AWELALGRLW DYLLWVQTLS DQVQEELLSS QISQELT
	NLMDETMKEV KAYKSELEAQ LSPMAEETQA RLHKELQAAQ ARLGADMEDV RSRLAQYRGE
	VQAMLGHSTE ELRARLASHL RKLRKRLLRD AEDLQKRLAV YQAGASEGAE RGVSAIRERL
	GPLLEQGRLR AATVGSLAGQ PLRERAQSLG TRLRGRLEEV GMQARDRLDE VREQMEEVRA
	KVEQQA‐ ‐ ‐ ‐ ‐ ‐AEVFQARL KSWFEPLVED MQRQWAGLVE KVQATMGVSA TPVPSDNH
Geomyidae	**Botta's pocket gopher** (*Thomomys bottae*; JANJXW010000039)
	32813 Da
	MKLLW AVLAA TLLAG CQA
	ELEPEVEVLE RSPWPASQPW ELALSRFWDY LRWVQTLSDQ VQEELLSSQV TQELT
	VLMEETIKEV KAYKSEVESQ LSPMAVETQA RLNKELQAAQ ARLEADMEDV RTRLAQYQAE
	VQTMLGQSTE EMRARLASHL RKLRKRLSRD AEDLQKRLAV YRAGAHEGAE RGVSAIRERL
	GPLVEQGRTR AASLDASVGK PLRDRAQALG TRLRGRLEEV GSQARSRLEE VRTQMEEVRA
	KVEQQ‐‐ ‐ ‐ ‐ ‐ ‐AQAFQSRL KSWFDPLVED MQRQWAELVE KMQTAVGTNA VPVPATDNH
	**Plains pocket gopher** (*Geomys bursarius*; JANJXW010000039)
	32921 Da
	MKLLW AVLAA TLLAG CQA
	ELEPEPEVLE RSPWQASQPW ELALSRFWDY LRWVQTLSDQ VQEELLSSQV TQELT
	VLMEETMKEV KAYKSEVESQ LSPMAVETQA RLNKELQAAQ ARLEADMEDV RTRLAQYQAE
	AQTMLGQSTE EMRARLASHL RKLRKRLSRD AEDLQKRLAV YRVGAREGAE RGVSAIRERL
	GPLVEQGRTR AASLDSSLGK PLRDRAQALG ARLRGRLEEV GSQARDRLEE VRTQMEEVRA
	KVEQQ‐ ‐ ‐ ‐ ‐ ‐ ‐AQAFQSRL KSWFEPLVED MQRQWAELVE KMQTAVGTNA VPVPATDNH
Heteromyidae	**Banner‐tailed kangaroo rat** (*Dipodomys spectabilis*; JAHHPX010000266)
	33146 Da
	MKLLWAVLVV TLLAG CRA
	DVVEPESEPE MLERSQWQAS HPWELALGRF WDYLRWVQTL SDQVQEELLS SQVTQELT
	VLMEETMKEI KAYKSEVESQ LSPMAAETQA RLNKELQAAQ ARLEADMEDV RARLAQYRTE
	VQTMLGQSTE EMRARLASHL RKLRKRLGRD AEDLQKRLAV YEAGAHEGVE RSVSALRERL
	GPLVEQGRTR AAGLDAGAAR LRDRAQALGA RLRGRLEEVG SQTRDRLEEV RVQMEEVRAK
	MEQQ‐ ‐ ‐ ‐ ‐ ‐ ‐AQAFRSRLK SWFEPLVEDM QQYWAELVEK MQAAVGPNAV PVPAIDNH
	**Stephen's kangaroo rat** (*Dipodomys stephensi*; PVHN010014934)
	33108 Da
	MKLLW AVLVV TLLAG CRA
	DMVEPESEPE MLERSQWQAS HPWELALGRF WDYLRWVQTL SDQVQEELLS SQVTQELT
	VLMEETMKEI KAYKSEVESQ LSPMAAETQA RLNKELQAAQ ARLEADMEDV RARLAQYRTD
	VQTMLGQSTE DMRARLASHL RKLRKRLGRD AEDLQKRLAV YGAGAHEGVE RSVSALRERL
	GPLVEQGRTR AAGLDAGAAR LRDRAQTLGA RLRGRLEEVG SQTRDRLEEV RVQMEEVRAK
	MEQQ‐ ‐ ‐ ‐ ‐ ‐ ‐AQAFRSRLK SWFEPLVEDM QQYWAELVEK MQAAVGPNAV PVPAIDNH
	**Merriam's kangaroo rat** (*Dipodomys merriami*)
	33060 Da
	MKLLW AVLVV TLLAG CRA
	EVVEPESEPE MLERSQWQAS HPWELALGRF WDYLRWVQTM SDQVQEELLS SQVTQELT
	VLMEETMKEI KAYKSEVESQ LSPMAAETQA RLNKELQAAQ ARLEADMEDV RARLAQYRTD
	VQTMLGQSTE EMRARLASHL RKLRKRLGRD AEDLQKRLAV YGAGAHEGVE RSVSALRERL
	GPLVEQGRTR AAGLDAGAAR LRDRAQALGA RLRGRLEEVG SQTRDRLEEV RVQVEEVRAK
	MEQQ‐ ‐ ‐ ‐ ‐ ‐ ‐AQAFRSRLK SWFEPLVEEM QQYWAELVEK MQAAVGPNAV PVPAVDNH
	**Pacific pocket mouse** (*Perognathus longimembris pacificus*; RJWR010000410)
	33012 Da
	MKILWAVLVV TLLAG CQA
	ELEPELEPEV PERSPWQAGQ PWEQALSRFW DYLRWVQTLS DQVQEELLSS QVTQELT
	ALMEETMKEV KAYKSEVESQ LSPMAVETQA RLNKELQAAQ ARLEADMEDV RTRLAQYRTE
	VQTMLGQSTE EMRARLASHL RKLRKRLSRD AEDLQKRLAV YGAGAREGAE RGVSAIRERL
	GPLVEQGRVR AAGLDAGAAQ PLRDRAQALG TRLRGRLEEV SSQARDRLEE VRVQMEEVRA
	KVEQQ‐ ‐ ‐ ‐ ‐ ‐ ‐AEAFQSRL KSWFEPLVED MQRQWAELVE KMQAALGTNA VPVPAIDNH
Human, Mouse and Marmot apoE Data	
	**Human** (*Homo sapiens*; P02649)
	34290 Da
	MKVLW AALLV TFLAG CQA
	KVEQAVETEP EPELRQQTEW QSGQRWELAL GRFWDYLRWV QTLSEQVQEE LLSSQVTQEL R
	ALMDETMKEL KAYKSELEEQ LTPVAEETRA RLSKELQAAQ ARLGADMEDV RGRLVQYRGE
	VQAMLGQSTE ELRVRLASHL RKLRKRLLRD ADDLQKRLAV YQAGAREGAE RGLSAIRERL
	GPLVEQGRVR AATVGSLAGQ PLQERAQAWG ERLRARMEEM GSRTRDRLDE VKEQVAEVRA
	KLEEQAQQIR LQAEAFQARL KSWFEPLVED MQRQWAGLVE KVQAAVGTSA APVPSDNH
	**Mouse** (*Mus musculus*; PO8226)
	33968 Da
	MKALWAVLLV TLLTG CLA
	EGEPEVTDQL EWQSNQPWEQ ALNRFWDYLR WVQTLSDQVQ EELQSSQVTQ ELT
	ALMEDTMTEV KAYKKELEEQ LGPVAEETRA RLGKEVQAAQ ARLGADMEDL RNRLGQYRNE
	VHTMLGQSTE EIRARLSTHL RKMRKRLMRD AEDLQKRLAV YKAGAREGAE RGVSAIRERL
	GPLVEQGRQR TANLGAGAAQ PLRDRAQAFG DRIRGRLEEV GNQARDRLEE VREHMEEVRS
	KMEEQTQQIR LQAEIFQARL KGWFEPIVED MHRQWANLME KIQASVATNP IITPVAQENQ
	**Yellow‐bellied marmot** (*Marmota flaviventris*; QZWP02023227)
	34177 Da
	MKVLWAVLVT TLLAG CLA
	ELELEPEVQE QTNWQIGQTR QPWELALGRF WDYLHWVQTL SDQVQEELLS SQVTQELA
	VLIEDTMKEV KAYKSELEEQ LRPMAEETQA RLSKELQAAQ ARLGADMQDV RNRLEQYRSE
	VRAMVGQSTE ELRARLASHL RKLRKRLQRD AEDLQKRLAV YQAGAREGAE RGVSAIRERL
	GPLVEQGRLR AATVSSLASQ PLRERAQAWG ERLRGRLEEV GSRARDRLDE VREQVEEVRA
	KVEEQTAQMR LQAEAFQARL KSWFEPLVED MQRQWAGLVE KMQKAMGAST APAPSDNH

*Note*: Shown along with the molecular weight in Daltons (Da), the common name and the scientific name, is the accession number for the genomic entry from which the *APOE* gene was extracted.

^a^ Exon 2 was not found in these entries.

## RESULTS

With few exceptions, it was possible to locate the three coding exons of rodent apoE from the entries in the NCBI database. The resulting encoded sequences of the hystricomorphs and castorimorphs are listed in Table 1.

Hystricomorphs belong to either of two infraorders, *Hysticognathi* or *Ctenodactylomorphi*. The hystricognathous rodents were found to have two gaps in their encoded sequences when aligned with the human, house mouse, and yellow‐bellied marmot sequences. The first of these gaps ranged in size between five to three missing residues, as indicated in Figure 1. The missing five residues belong to the superfamily *Cavioidea*. All the others had a four‐residue gap, except for the pacarana (*Dinomys branickii*) that was missing three.

The other gap consisted of seven missing residues. As shown in Figure 2, this latter gap was in the same location for both the hystricognathous rodents and the castorimorphs. As can be seen in both figures, the gaps are bordered by conserved sequences.

Data were found only for one member of the infraorder *Ctenodactylomorphi*. The gundi APOE was an exception. It was missing 11 residues, as shown in Table 1.

## DISCUSSION

Mammalian apolipoproteins are encoded by three exons. In the case of APOE, the gene consists of four exons, the first being noncoding. The second exon, in almost all species, encodes the first 15 amino acids of the signal sequence. The terminal 3 amino acids are encoded by exon 3 along with the beginning sequence of the mature apolipoprotein. As the data in Table 1 indicate, all the rodents had an 18 amino acid signal sequence; however, the initial length of the N‐terminal region and the C‐terminal regions varied as reflected in the differences seen in Table 1. This is not unusual among mammals. In the house mouse, along with all other members of the *Muridae* family, the N‐terminal sequence encoded by exon 3 contains just 53 residues, and in the harbor seal (*Phoca vitulina*), the corresponding region contains 83 residues (Davis et al., 1991). Among the hystricognathous sequences reported in Table 1, the N‐terminal sequence contained 55 residues, except for the two members of the family *Hystricidae*. The Malayan and crested porcupines had the longest length for this region of APOE with 62 residues.

As for castorimorphs, the N‐terminal sequence encoded by exon 3 varied between 55 residues in the two gophers to 58 in the three kangaroo rats. In spite of these variations seen in both the castorimorph and the hystricognathous N‐terminal regions, the last 42 residues, encoded by exon 3, aligned quite well this region of human APOE.

As reported here, in the region encoded by exon 4, the hystricognathous rodents, in contrast to the castorimorphs, were missing residues corresponding to residues between 192 and 196 in the human APOE sequence.

Initially, the apolipoprotein E was called the arginine‐rich protein, aka ARP, (Shore et al., 1974; Shore & Shore, 1976). The region encoded by exon 4 of rodent *APOE* was also found to have a high content of arginine. Of the 21 residues containing arginine in the human sequence, 19 align with those in the hystricomorph and castorimorph sequences. A notable exception is the arginine at residue 61 in the human sequence that aligns with a threonine in these rodent sequences. Interestingly, other primates as well as most rodents have a threonine at this location. The change in primates from a threonine to an arginine occurred sometime between the divergence of bonobos and chimpanzees from the human lineage and the appearance of the Denisovans (Huebbe & Rimbach, 2017; McIntosh et al., 2012). Among rodents, there is an exception; those belonging to the tribe *Marmotini* have an alanine at this position. In Table 1, an example of this can be seen in the sequence of the yellow‐bellied marmot.

In reporting on the mammalian evolution of APOE, Yang et al. (1991) examined the sequences of eight mammals (bovine, canine, human, macaque, rabbit, and three rodents). The rodent sequences were those of the guinea pig, mouse, and rat. The aligned sequences were separated into a series of 14 helical regions. Except for two regions, the alignment was very good. The exceptions were E‐10, where the guinea pig was missing five residues, and E‐13, where the guinea pig was missing seven residues. The alignment showed that the first deletion was close to the region of human APOE that has been identified as the hinge region (residues 200–215), which enables the apolipoprotein to change confirmation after binding to lipids or a membranous surface of a cell (Chen et al., 2011; Marais, 2019). The E‐13 region and the beginning sequence of E‐14 region contain what was subsequently indicated to be the lipid‐binding region of APOE (residues 241–272 in the human sequence) (de Lima Pizzico et al., 2024). Considering that both hystricomorphs and castorimorphs lack amino acids that align with residues 247–253 in the human sequence, it is possible that the lipid‐binding region is even shorter, that is, between the very conserved segment consisting of residues from 254 to 272 in the human sequence.

## AUTHOR CONTRIBUTIONS

Dr. Don Puppione is the sole author of this paper.

## CONFLICT OF INTEREST STATEMENT

The author declares that he has no conflict of interest.

## ETHICS STATEMENT

No animal subjects were used in this research.
